# Characteristics of cases needing advanced treatment for intractable Posner–Schlossman syndrome

**DOI:** 10.1186/s12886-017-0438-y

**Published:** 2017-04-11

**Authors:** Kazuichi Maruyama, Yuko Maruyama, Sunao Sugita, Kazuhiko Mori, Yu Yokoyama, Shiho Sanuki-Kunimatsu, Hiroko Nakagawa, Shigeru Kinoshita, Manabu Mochizuki, Toru Nakazawa

**Affiliations:** 1 Department of Ophthalmology and Visual Science, Tohoku University Graduate School of Medicine, Sendai, Japan; 2 Department of Ophthalmology, Kyoto Prefectural University of Medicine, Kyoto, Japan; 3 RIKEN Center for Development Biology, Kobe, Japan; 4 Department of Ophthalmology, Tokyo Medical and Dental University, School of Medicine, Tokyo, Japan

**Keywords:** Posner–Schlossman syndrome (PSS), Corneal endothelial cell (CEC) density, Intraocular pressure (IOP), Glaucoma surgery, Polymerase chain reaction (PCR)

## Abstract

**Background:**

In Posner-Schlossman syndrome (PSS), which is characterized by recurrent unilateral attacks of ocular hypertension. Surgical treatment is sometimes necessary because intraocular pressure (IOP) cannot be controlled with anti-glaucoma medications. To identify the clinical features of Posner–Schlossman syndrome (PSS) indicative of the need for intraocular pressure (IOP)-controlling surgery.

**Methods:**

This study was a retrospective case-series analysis of the clinical charts of 33 patients diagnosed with PSS, who underwent surgery to control IOP or received medication only. Various clinical factors were compared between the surgical and medication groups.

**Results:**

The surgical group had a higher corneal endothelial cell (CEC) density loss (*p* < 0.05), higher maximum IOP (*p* < 0.01), greater visual field loss (*p* < 0.01) and higher positive number for cytomegalovirus (CMV) (*p* < 0.001) than the non-surgical group. Eighteen of the 33 patients had a high CEC reduction ratio. Of these 18, 16 required glaucoma surgery.

**Conclusions:**

PSS patients with a higher CEC reduction ratio, higher maximum IOP, greater visual field loss and higher positive number for CMV in the aqueous humor tended to be more likely to require progressive treatment, such as glaucoma surgery.

**Electronic supplementary material:**

The online version of this article (doi:10.1186/s12886-017-0438-y) contains supplementary material, which is available to authorized users.

## Background

Posner–Schlossman syndrome (PSS), also known as glaucomatocyclitic crisis, was first described in 1948 [1]. Patients have open angles but suffer from recurrent unilateral attacks of mild iritis with high intraocular pressure (IOP). During an attack, patients present with blurred vision and mild inflammation in the anterior chamber, with the development of small to medium-sized fine keratic precipitates. The attacks resolve spontaneously in a few days to a week, and the IOP is normal in the remission periods. The original report on PSS by Posner and Schlossman suggested that the attacks did not cause permanent damage to the eye, but subsequent reports raised questions on the benign nature of the disease [2, 3]. PSS typically occurs in younger individuals, making the prevention of vision loss caused by high IOP an important goal in disease management [3].

Treatment of PSS aims at controlling inflammation and elevated IOP. Frequent attacks of high IOP are particularly dangerous because they can easily affect vision by causing progressive visual field defects. Therefore, IOP control is the most important treatment goal, with a favoured initial approach that combines anti-inflammatory and anti-glaucoma eye drops. However, some patients are not responsive to medical treatment and must undergo glaucoma surgery to prevent visual field loss. In some of these cases, the effectiveness of trabeculectomy has been reported [4]. In general, however, only a subset of PSS patients with progressive visual field defects need to undergo glaucoma surgery.

Since its first description, the etiology of PSS has been the subject of debate. Recently, some cases have been associated with viral infections, such as cytomegalovirus (CMV), herpes simplex virus and varicella zoster virus [5–7]. Polymerase chain reaction (PCR) analysis of the aqueous humor of the affected eye of PSS patients has shown that 52% were positive for CMV [6]. Other recent studies have shown that in some PSS patients, the corneal endothelial cell (CEC) density is lower in the affected eye than in the healthy eye [8], and that some of these cases have related CMV infections [6].

The purpose of the present study was to identify the clinical features of PSS patients who needed glaucoma surgery. Our data showed that PSS patients who underwent glaucoma surgery had higher IOP, more frequent ocular attacks, a poorer visual field, lower CEC density and a greater incidence of CMV in the aqueous humor.

## Methods

### Patients

This study examined a retrospective, consecutive case series of patients diagnosed with PSS at the glaucoma and uveitis clinics of Tohoku University and Kyoto Prefectural University of Medicine between 2005 and 2014.

**Fig. 1:**
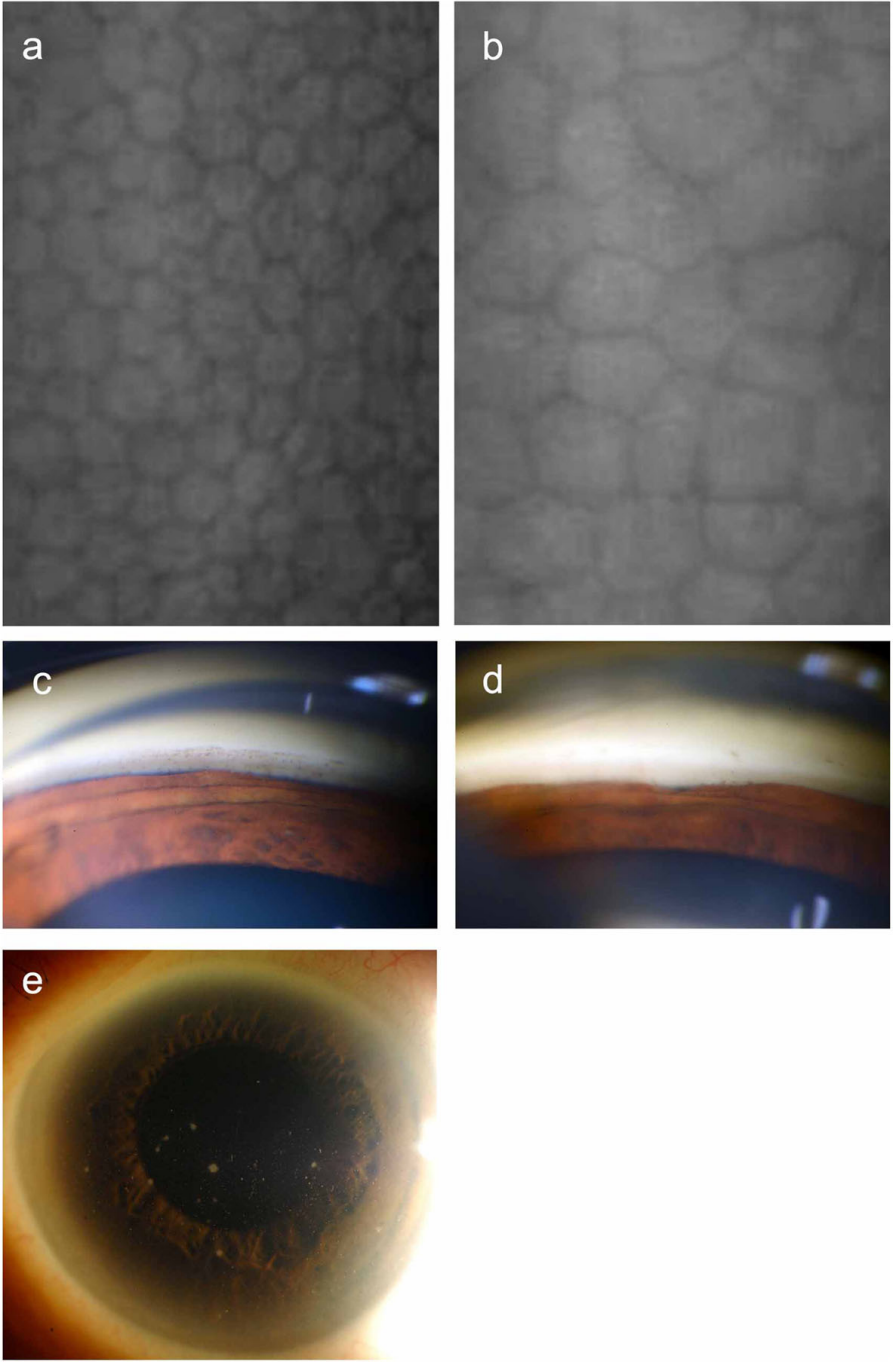
Specular microscope photographs of (**a**) a non-affected eye and (**b**) an affected eye. Gonioscopic slit lamp photographs of (**c**) a non-affected eye and (**d**) an affected eye. **e** Slit lamp photograph of the anterior segment of an affected eye (a white precipitate is visible on the corneal endothelial side)

Thirty-three eyes of 33 PSS patients (15 females and 18 males, mean age: 51.8 ± 18.9 years) were included. The diagnosis of PSS was made on the basis of previously described clinical features of the disease, (Fig. 1) [3] including mild unilateral anterior uveitis accompanied by high IOP, small white keratic precipitates mainly on the endothelial surface of the central cornea, no posterior synechia and no inflammatory lesions in the posterior segment of the eye. Although inflammation in the anterior chamber of PSS patients generally resolves in 2 to 3 weeks with the topical application of steroids, the patients experience recurrences of anterior uveitis with high IOP. Other etiologies of uveitis in the patients included here were ruled out with a routine systemic investigation that included a tuberculin skin test, chest X-ray, serum angiotensin-converting enzyme test and serological tests for syphilis, toxoplasmosis and HTLV-1.

### Study Design

The clinical data of the patients, including their age, maximum recorded IOP, central CEC density, attack frequency and history of glaucoma surgery, were collected from their clinical charts.

The patients were divided into two groups on the basis of their need for glaucoma surgery. Twenty-one patients underwent filtration surgery because they did not respond to IOP-reducing medications and had progressive visual field loss, whereas 12 patients (7 females and 5 males, mean age: 45.3 ± 23.7 years) did not undergo surgery. The 21 surgical cases (7 females and 14 males, mean age: 55.5 ± 14.9 years) underwent trabetrabeculectomy with mitomycin in 17 patients, trabeculotomy in 2 patients and trabecutome in 2 patients after receiving a full course of treatment with IOP-reducing medications.

After the data collection, clinical factors, including visual acuity (VA), frequency of ocular inflammatory attacks, visual field data obtained with the Humphrey field analyser (HFA II-I, Zeiss, Germany), maximum IOP as measured with a Goldmann applanation tonometer and CEC density, were compared between the two groups.

### PCR analysis

An aliquot (0.1 ml) of the aqueous humor was taken from the anterior chamber of the PSS patients at the time of high IOP phase with inflammation, as well as the time of surgery in the surgical group. These samples were processed and analysed with a multiplex PCR system to identify genomic DNA of the human herpes virus, as described previously [9, 10].

### CEC density measurement and classification of the patients

The central CEC density of the affected eye of the PSS patients was measured with a specular microscope (EM-3000, TOMEY Corporation, Nagoya, Japan) at the initial presentation in all patients and at the following time points in the patients who underwent surgery: before surgery and at 3, 6, 9, 12, 18, 24, 36 and 48 months after surgery. CEC density was measured in the contralateral healthy eye and used as a control. The CEC reduction ratio was calculated as follows: (CEC density of the control eye − CEC density of the affected eye)/CEC density of the control eye × 100 (%).

We divided the total group of patients into low and high CEC reduction ratio groups according to a cut-off point determined with a receiver operator curve (ROC) space analysis. We then used Fisher’s exact test to compare the number of patients requiring surgery in these groups. All analyses were performed with Prism software (version 5.0.1, Graph Pad Software, San Diego, CA).

### Statistics and mathematical analysis

A Student’s *t*-test was used to analyse the maximum IOP, final mean deviation of the visual field (before surgery), CEC density and CEC reduction rate. Mann–Whitney test was used to analyse attack frequency. Fisher’s exact test was used to analyse the number of patients who required surgery. *P*-values of <0.05 were considered statistically significant. These analyses were also performed with Prism software.

## Results

### Patient details and results of the clinical examination

**Fig. 2:**
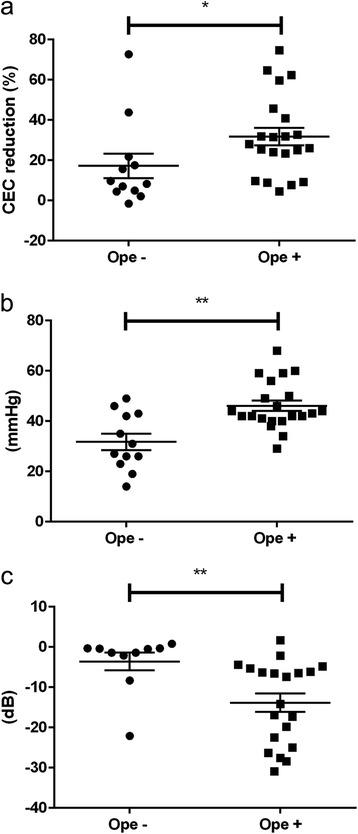
**a** CEC reduction (%) in the non-surgical and surgical groups (*p < 0.05). **b** Comparison of the maximum IOP in the non-surgical and surgical groups (**p < 0.01). **c** Comparison of the Humphrey visual field analyser-measured mean deviation in the non-surgical and surgical groups (**p < 0.01)

This study included 33 affected eyes and 33 unaffected contralateral eyes of 33 patients. Table 1 shows all data for the patients, including the PCR results (Additional file 1 shows more detailed data for the patients). A comparison of CEC reduction in the two groups showed that it was significantly higher in the surgical group (31.8 ± 4.4%) than in the non-surgical group (17.2 ± 6.1%) (*p* < 0.05) (Fig. 2a). The maximum IOP was 46.1 ± 2.1 mmHg in the surgical group and 31.8 ± 3.2 mmHg in the non-surgical group, indicating a significant difference (*p* < 0.01). The HFA-measured mean deviation (MD) was −13.9 ± 2.3 dB in the surgical group and −3.6 ± 2.2 dB in the non-surgical group, indicating a significant difference (*p* < 0.01) (Fig. 2b, c).

**Table 1:** Detailed list of patients

Case	Gender	Affected eye	Reduction rate (%)	Highest IOP (mmHg)	Pre Treat VA (log MAR)	Final MD (dB)	PCR	Ope
1	M	L	-1.5	31	-0.1	NP	−	−
2	F	R	4.4	35	0.1	NP	−	−
3	F	R	7	14	-0.1	−0.32	−	−
4	F	R	9.7	27	0.2	NP	−	−
5	M	L	15.6	19	1	GP	−	−
6	M	R	21.8	23	−0.2	-8.33	CMV	−
7	F	R	43.8	46	0.1	−0.4	−	−
8	F	R	4.9	42	−0.1	−1.37	−	−
9	M	R	72.7	26	0	−0.28	−	−
10	M	R	8.3	43	−0.2	−1.43	−	−
11	F	L	2.2	49	−0.1	−0.46	−	−
12	F	L	17.6	26	−0.2	0.8	−	−
13	M	R	9.1	68	0.4	−28.38	−	+
14	F	L	23.3	68	−0.2	-6.45	CMV	+
15	M	R	23.9	68	−0.1	−4.38	−	+
16	F	R	25	68	0.3	−2.2	−	+
17	M	R	31.9	68	0	−4.76	−	+
18	M	R	31.9	68	0.1	−7.4	−	+
19	M	L	31.6	68	-0.1	GP	−	+
20	F	L	40.8	68	-0.1	1.67	Parvo B19	+
21	F	L	59.6	68	0	−5.25	−	+
22	F	L	74.6	68	0.5	−6.31	−	+
23	F	R	7.8	68	0	−16.9	−	+
24	M	R	32.6	68	0	-6.17	CMV	+
25	M	R	4.4	68	−0.2	−26.27	−	+
26	F	R	28	68	0	-19.82	CMV	+
27	M	R	8.8	68	0.5	-30.85	CMV	+
28	M	L	45.6	42	−0.2	−27.65	−	+
29	F	L	64.7	42	−0.1	-6.57	CMV	+
30	M	L	9.7	34	0.2	-24.98	CMV	+
31	M	R	25.3	44	−0.2	−14.16	−	+
32	M	R	62.3	46	0	17.34	−	+
33	M	L	26	43	0.4	−22.478	−	+

*IOP* intraocular pressure; *VA* visual acuity; *MD* mean deviation; *M* male; *F* female; *NP* not performed; *GP* Goldmann perimetry; *CMV* cytomegalovirus; *TLE* trabeculectomy; *TLO* trabeculotomy; *TTO* trabecutome

**Fig. 3:**
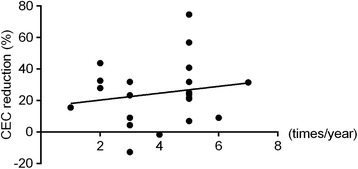
Correlation between reduced CEC density and attack frequency (*r* = 0.17, *p* = 0.46)

Previous reports showed that the CEC density is lower in the affected eyes of PSS patients than in the contralateral, unaffected eyes because of the repeated occurrence of attacks of high IOP [8]. We therefore investigated the relationship between reduced CEC density and attack frequency, but we did not find a significant correlation (*r* = 0.17, *P* = 0.46) (Fig. 3). We also investigated the correlation between CEC density and other factors, such as maximum IOP and final MD. No correlation was found in any of these comparisons (data not shown).

### Relationship between CEC density and frequency of glaucoma operation

**Fig. 4:**
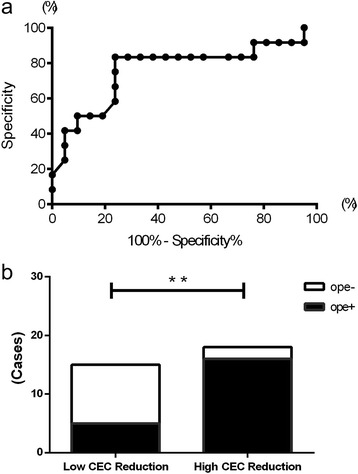
**a** Receiver operator curve (ROC) space for reduced CEC density. **b** Comparison of the number of patients who required filtration surgery in the high CEC reduction ratio CEC density and low CEC reduction ratio groups (**p < 0.01)

Next, we drew the ROC space for all 33 patients to determine the cut-off point for separating them into the low and high CEC reduction ratio groups. This analysis showed that the most optimal cut-off point was a CEC reduction ratio of 22.6% (Fig. 4a). We then compared the proportion of patients in the resulting two groups that required glaucoma surgery. We found that 88.9% (16/18) of the patients in the high CEC reduction ratio density group required glaucoma surgery, whereas 33.3% (5/15) of the patients in the low CEC density group required surgery. This result indicated a significant difference (*p* < 0.01) (Fig. 4b).

### PCR examination of aqueous humor samples

We next investigated pathogenic DNA in the aqueous humor of the patients and compared the results in the surgical and non-surgical groups (Table 1). Seven patients were positive for CMV, and only one patient was positive for *parvovirus B19* virus DNA in the aqueous humor. CMV DNA was found in 6 of the 21 surgical patients, but only in 1 of the 12 non-surgical patients. Thus, the surgical patients had positive results for CMV DNA in the aqueous humor significantly more often (*p* < 0.001).

## Discussion

In this study, we examined the clinical characteristics of PSS patients who underwent glaucoma surgery compared with patients who did not need surgery. Our data showed that the surgical-requiring group of patients had higher maximum IOP, greater visual field loss and higher rate of CMV infection than the non-surgical group. Importantly, we also found that the surgical group had a higher CEC reduction ratio density than the non-surgical group. Thus, these clinical features can be considered as indicators of the need for glaucoma surgery in PSS patients. This type of surgery is usually indicated for patients with primary open-angle glaucoma and medically uncontrollable high IOP. Thus, high IOP in PSS patients is a clear indicator that advanced treatment is needed to prevent vision loss.

A key finding of this study was that patients requiring surgery had a reduced CEC density. A number of previous investigators have suggested that frequent attacks of high IOP in PSS and CEC density may be related [8]. A previous report showed that the CEC density was lower in the affected eyes of PSS patients than in the unaffected eyes, and concluded that a difference of over 20% in endothelial cell density was a result of frequent attacks of high IOP [8]. Therefore, the duration of high IOP attacks may be correlated with the severity of the disease.

We found in this study that a CEC reduction ratio of 22.6% (compared with that in the unaffected eye) was the cut-off point for the need for surgical treatment. To investigate the importance of CEC density in more detail, we used this cut-off point to divide the patients into low and high CEC density groups. Interestingly, we found that almost 90% of the patients in the high CEC density group needed surgical treatment. This prompted us to investigate in detail the pathological conditions in the aqueous humor of the group with a high CEC reduction ratio.

The hallmark of PSS is recurrent episodes of self-limiting, mild, non-granulomatous anterior uveitis with markedly elevated intraocular pressure [11, 12]. The development of glaucomatous damage from PSS is also a well-known feature of the condition [3, 13]. Although the etiology of PSS is still controversial, recent reports have shown that CMV plays a pathogenic role in some PSS patients [7, 14]. CMV was previously reported to have an affinity for CECs and the trabecular meshwork [15]. When CMV infection occurs in the trabecular meshwork, the friction of the passage of the aqueous humor is believed to increase. This suggests that in at least some patients, severe CEC reduction and high IOP attacks may be caused by CMV infection. In the current study, CMV DNA was detected in 28% of the surgical patients (6 of 21). On the other hand, CMV was detected in only 8% of the non-surgical patients (1 of 12). Therefore, CMV may be related to high IOP attacks, including anterior inflammation, in the surgical treatment group.

This study also had limitations because of the study design and sample size. However, even with the small number of patients, our data clearly showed that the surgical group had a number of clinical features that distinguished it from the non-surgical PSS group.

## Conclusions

This study found that PSS patients with a high CEC reduction ratio required glaucoma surgery more often than those with a low CEC reduction ratio. A high CEC reduction ratio in the affected eye of PSS patients might thus indicate a need for close observation and progressive treatment.

## Additional file

Additional file 1: The detailed data for the patients. CEC: corneal endothelial cell; IOP: intraocular pressure; VA: visual acuity; MD: mean deviation; M: male; F: female; L: left; R: right; NP: not performed; GP: Goldmann perimetry; PCR: polymerase chain reaction; CMV: cytomegalovirus; TLE: trabeculectomy; TLO: trabeculotomy; TTO; trabecutome. (XLSX 43 kb)

## Abbreviations

CEC: Corneal endothelial cell
CMV: Cytomegalovirus
IOP: Intraocular pressure
PCR: Polymerase chain reaction
PSS: Posner–Schlossman syndrome
ROC: Receiver operator curve
